# Small chromosomes among Danish *Candida glabrata* isolates originated through different mechanisms

**DOI:** 10.1007/s10482-013-9931-3

**Published:** 2013-05-14

**Authors:** Khadija Mohamed Ahmad, Olena P. Ishchuk, Linda Hellborg, Gloria Jørgensen, Miha Skvarc, Jørgen Stenderup, Dorte Jørck-Ramberg, Silvia Polakova, Jure Piškur

**Affiliations:** 1 Department of Biology, Lund University, Sölvegatan 35, 223 62 Lund, Sweden; 2 BioCentrum-DTU, Technical University of Denmark, 2800 Lyngby, Denmark; 3 Department of Clinical Microbiology, Regionshospitalet Herning, 7400 Herning, Denmark

**Keywords:** Phylogeny, Pathogenic yeast, Chromosome, Genome rearrangements

## Abstract

We analyzed 192 strains of the pathogenic yeast *Candida glabrata* from patients, mainly suffering from systemic infection, at Danish hospitals during 1985–1999. Our analysis showed that these strains were closely related but exhibited large karyotype polymorphism. Nine strains contained small chromosomes, which were smaller than 0.5 Mb. Regarding the year, patient and hospital, these *C. glabrata* strains had independent origin and the analyzed small chromosomes were structurally not related to each other (i.e. they contained different sets of genes). We suggest that at least two mechanisms could participate in their origin: (i) through a segmental duplication which covered the centromeric region, or (ii) by a translocation event moving a larger chromosome arm to another chromosome that leaves the centromere part with the shorter arm. The first type of small chromosomes carrying duplicated genes exhibited mitotic instability, while the second type, which contained the corresponding genes in only one copy in the genome, was mitotically stable. Apparently, in patients *C.* *glabrata* chromosomes are frequently reshuffled resulting in new genetic configurations, including appearance of small chromosomes, and some of these resulting “mutant” strains can have increased fitness in a certain patient “environment”.

**Electronic supplementary material:**

The online version of this article (doi:10.1007/s10482-013-9931-3) contains supplementary material, which is available to authorized users.

## Introduction

Yeasts are unicellular eukaryotic organisms, and several species have been reported as opportunistic human pathogens. *Candida glabrata* has for many years been known to represent non-pathogenic normal flora in healthy humans (Stenderup and Pederson 1962). This yeast can be abundant in relatively healthy individuals, but it also causes vaginal candidiasis, which is a common mucosal infection that occurs in healthy, immuno-competent women (Mentel et al. 2006) and even systemic infections. The mortality rate of systemic infections caused by *C. glabrata* is high as they are difficult to treat because of *C. glabrata* resistance to many antifungal drugs (Hitchcock et al. 1993; Komshian et al. 1989; Willocks et al. 1991). Because of both, the increased use of immunosuppressive therapy and also the prolonged use of wide spectrum antibiotics, during the last years the number of systemic and mucosal infections with *C.* *glabrata* has increased. This yeast has been reported to be the second most frequently found opportunistic yeast in humans, just after *Candida albicans* (Fidel et al. 1999).

*C. glabrata* is a rather close relative of *Saccharomyces cerevisiae*, and the two yeasts separated after the yeast whole genome duplication (WGD), app. 50 million years ago, both species are distant relatives of *C. albicans* (Dujon et al. 2004). Unlike the dimorphic diploid yeast *C.* *albicans*, all isolates of *C. glabrata* so far seem to be haploid. Mating in *C. glabrata* has not yet been observed and so this yeast is apparently asexual (Kaur et al. 2005). *C. glabrata* has been reported to exhibit high karyotype variability and may undergo rapid genome reorganisation even during infection in patients (Shin et al. 2007; Muller et al. 2009). It has also been reported that independent isolates from the same patient having *C. glabrata* fungemia had different karyotype patterns (Klempp-Selb et al. 2000). Chromosomal rearrangements and aneuploidy in *C. albicans* and *C. glabrata* have been demonstrated to increase the virulence potential and particularly drug resistance (Selmecki et al. 2006; Poláková et al. 2009). On the other hand, chromosomal aneuploidy in multicellular eukaryotes (e.g. humans) is usually associated with some genetic disorders, for instance with cancer.

The formation of new chromosomes as a molecular mechanism which can increase virulence has been reported in our recent analysis of forty pathogenic strains (Poláková et al. 2009). Two reported strains had extra chromosomes with the size under 500 kb and we therefore named them as small or mini-chromosomes. The origin of the two discovered small chromosomes has been explained through segmental duplication over the centromeric regions. One small chromosome has been shown to be responsible for the increased resistance towards anti-fungal drug fluconazole. The duplicated segment encodes the ATP-binding cassette family (ABC) transporter and the observed gene duplication apparently elevated the resistance towards azole in the patient (Poláková et al. 2009).

In this study, we examined 192 isolates of *C. glabrata*, which had been collected from Danish patients during 1985–1999. The phylogenetic relationship was estimated and the strain karyotypes determined. Interestingly, new small chromosomes were found. One of our aims was to deduce the mechanism(s) which led to the origin of these small chromosomes, and another to find any possible connections between the genes on the small chromosomes and the strain phenotype.

## Materials and methods

### Clinical isolates

During 1985–1999 putative *C. glabrata* isolates from patients hospitalized at Danish hospitals were collected, mainly isolated from blood and involved in systemic infections, and deposited to the State Serum Institute (Copenhagen, Denmark). All other reported publicly available collections of pathogenic *C. glabrata* strains are based on much later samplings (see for example, Klempp-Selb et al. 2000). Thus, our collection represents a unique tool to study the early appearance and development of systemic infections with this yeast. These clinical isolates were in 2004 transferred to our laboratory at Lund University (the Piskur yeast collection). Each strain was isolated from a different patient, with exception of a few strains isolated from the same patient at different time periods. All available details on the strains and their isolation source are presented in Supplementary materials Table S1. Forty of the deposited strains have been analyzed previously (Poláková et al. 2009), and hereby we characterized the remaining collection, 152 strains.

### DNA extraction and polymerase chain reaction (PCR)

The yeast strains were grown overnight in YPD media (1 % yeast extract, 2 % Bacto Peptone and 2 % glucose) at 25 °C on rotary shaker. Genomic DNA was extracted according to the protocol described in Philippsen et al. (1991). Two regions, the nuclear 26S ribosomal DNA D1/D2 domain and a fast evolving intergenic spacer region (IGS, located between the nuclear *CDH1* and *ERP6* genes on chromosome A) were amplified using a Stratagene Robo-cycler. The nuclear 26S r D1/D2 domain was amplified with the primers: NL1 (5′-GCA TAT CAA TAA GCG GAG GAA AAG-3′) and NL4 (5′-GGT CCG TGT TTC AAG ACG G-3′) using the following conditions. First cycle with initial denaturation temperature 94 °C for 3 min, followed by 35 cycles of 94 °C for 2 min, 54 °C for 1 min and 72 °C for 2 min, completed by a final elongation at 72 °C for 5 min. The primers used to amplify the IGS locus were: “00605” (5′-C TCA CAA ATG GAT TCC TTA AAG AGT TCG -3′) and “00627” (5′-GT C ACC AGA GTT GGA GTA CAT GTA G-3′). The following conditions were applied. The initial denaturation at 94 °C for 3 min, followed by 35 cycles of 94 °C for 45 s, 52 °C for 1 min and 72 °C for 1 min, completed by a final denaturation at 72 °C for 5 min. The PCR products were purified with the QIAquick gel extraction kit (Qiagen, Dorking, UK). Concentration of DNA was measured using a NanoDrop ND-1000 spectrophotometer and the sequencing was performed by MWG Biotech (Germany).

### Sequence analysis and phylogenetic relationship

The obtained sequences were deposited in the GenBank and the accession numbers can be found in Supplementary materials Table S1. The sequences used for phylogenetic trees were based on the D1/D2 domain and the IGS locus and were analyzed and aligned using the BioEdit/ClustalW program **(**Thompson et al. 1994 **)**. All positions containing gaps and missing data were eliminated from the dataset (Complete deletion option) and there were a total of 489 for D1/D2, and 474 positions for IGS in the final dataset. The analysis approach followed the previously published one (Poláková et al. 2009), where we analyzed the first forty strains. The evolutionary history was inferred using the Neigbour-joining method (Saitou and Nei 1987) and the evolutionary distances were computed using the the maximum composite likehood method (Tamura et al. 2004). The evolutionary history was also inferred using Maximum Parsimony method. Phylogenetic and molecular evolutionary analyses were conducted using *MEGA* version 5 (Tamura et al. 2011).

### Azole susceptibility test

The yeast strains were inoculated into 5 ml YPD and grown overnight. The cells were pelleted and washed twice with sterile water. Yeast strains were spotted as 3 μl at different serial dilutions (10^3^, 10^4^, 10^5^, 10^6^ cells/ml) to obtain single cell colonies, using a lab hedgehog distributer on solid YPD medium with different concentrations of fluconazole (15, 45, 80, 125, 388.8, 1116.8 μg/ml). The plates were incubated for 48 h at 37 °C and then inspected visually for the appearance of single cell colonies at lower dilutions. Fluconazole was purchased from Toronto Research Chemicals (TRC), and the stock solutions were diluted in DMSO.

### Karyotypes and pulse-field gel electrophoresis (PFGE)

The chromosomes from each yeast isolate were prepared as described before (Petersen et al. 1999) and separated by pulse-field gel electrophoresis using a CHEF Mapper XA (Bio-Rad). The best separation was obtained under the following conditions: step 1, 240 s pulse for 6 h; step 2, 160 s pulse for 13 h; step 3, 120 s pulse for 10 h; step 4, 90 s pulse for 10 h and step 5, 60 s pulse for 3 h. The included angle was 60 with voltage 4.5 V/cm. The sequenced *C. glabrata* CBS 138 (Supplementary material Fig. S1) and *S. cerevisiae* S288c (Y1307) strains were used as the standards.

### Southern blotting

Chromosomes were separated by pulse-field gels, which were subsequently depurinated for 20 min by 0.25 M HCl, denaturated for 30 min (1.5 M NaCl; 0.5 M NaOH) and neutralized for 20 min (1.5 M NaCl; 1 M Tris–HCl, pH 7.5). The chromosomes were transferred to a Hybond-XL membrane (GE Healthcare) in 20 × SSC solution (1.5 M NaCl; 0.15 M sodium citrate) for 3–4 h by vacuum transfer (VacuGene^TM^XL). UV light was used to crosslink the transferred DNA fragments. Thirteen isotope labeled DNA probes, originating from genes in the vicinity of the thirteen known centromeres, were prepared using the sequenced *C. glabrata* strain CBS 138 as the template. The corresponding probes are listed in Supplementary materials (Table S2; Fig. S1, S2, S3 and S4). The following PCR conditions were used to amplify hybridization probes; initial denaturation at 94 °C for 3 min, followed by 35 cycles of 94 °C for 45 s, at 56 °C for 45 s, and 72 °C for 1 min, completed by a final denaturation at 72 °C for 5 min. PCR-products were purified using the QIAquick PCR purification kit (Qiagen). For membrane hybridization, 100 ng of the purified PCR product was diluted and used for [α^32^ P] dCTP labeling (GE Healthcare, Amersham rediprime^Tm^ II DNA labeling system) for 30 min at 37 °C. G-50 columns (GE Healthcare) were used to remove unincorporated nucleotides. The membrane was hybridized with 0.25 M Na_2_HPO_4_, 7 % SDS and 1 mM EDTA at 60 °C overnight. The membrane was washed twice with 2 % SDS in 100 mM Na_2_HPO_4_ at room temperature for 5 min and once at 60 °C for 20 min. Imaging Screen-K (35 × 43, Bio-Rad) and a personal Imager FX (Bio-Rad) were used to detect the hybridization signals. The membrane was stripped twice using boiled 0.1 % SDS for 5 min and used for re-hybridization with a new probe. Similarly, also several putative resistance genes were labeled and their presence on the small chromosomes analyzed.

### Small chromosomes stability test

In order to check the stability of the newly described small chromosomes, single colonies from the strains with small chromosomes were inoculated in 2 ml YPD and incubated overnight at 25 °C. 2 μl of the overnight culture was re-inoculated into a new 2 ml liquid YPD. After 70 generations, different dilutions were made for each individual strain, plated on YPD and incubated overnight at 25 °C. Eight to ten single colonies from each experiment were analyzed by PFGE.

### Quantification of the expression potential by RT-qPCR

The genes which were analyzed in the transcription studies are presented in Table 2. The yeasts used in the transcription study were grown in YPD with the supplement of glucose (20 g/l) as carbon source, and the RNA preparation and RT-qPCR analysis followed the method presented in Rozpedowska et al. (2011). 1 μg of RNA was used for the synthesis of cDNA using the SuperScript III Reverse Transcriptase kit with RNaseOUT Ribonuclease Inhibitor and random primers. The expression studies were carried out using SYBR GreenER qPCR SuperMix with the cDNA as a template and the specific primers. All kits and compounds were obtained from Invitorgen. The PCRs were run as duplicates in RotorGene 2000 cycler under the conditions specified by Invitrogen. The take off and the amplification values, obtained from the relative quantification performed using the RotorGene 2000 software, were used to quantify the expression ratios with the help of REST 2009 V2.0.13 with RG mode25. The β-actin gene was treated as endogenous reference, and we used the sequenced strain Y1092 (CBS 138) as untreated strain for comparison.

## Results and discussion

### Phylogenetic relationship

**Fig. 1:**
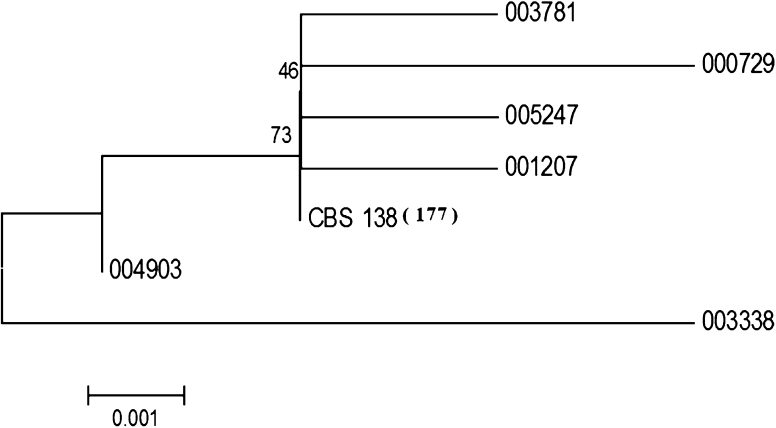
Phylogenetic relationships among pathogenic *C. glabrata* strains, based on seven different haplotypes, as deduced by Neighbor-joining method. The analysis is based on the D1/D2 domain of the 26 rDNA encoding locus. The numbers correspond to the museum numbers of the initial collection and can be found in Supplementary materials Table S1. Among the analyzed sequences (for accession numbers see Table S1), which had the 489 positions, 177 were identical with the *C. glabrata* type strain CBS 138. The strains belonging to the same haplotype are described in Supplementary materials Table 1. The bootstrap values are shown on some branches and the tree was not rooted. The *scale bar* in Neigbour-joining analysis corresponds to 0.001substitution per nucleotide site

**Fig. 2:**
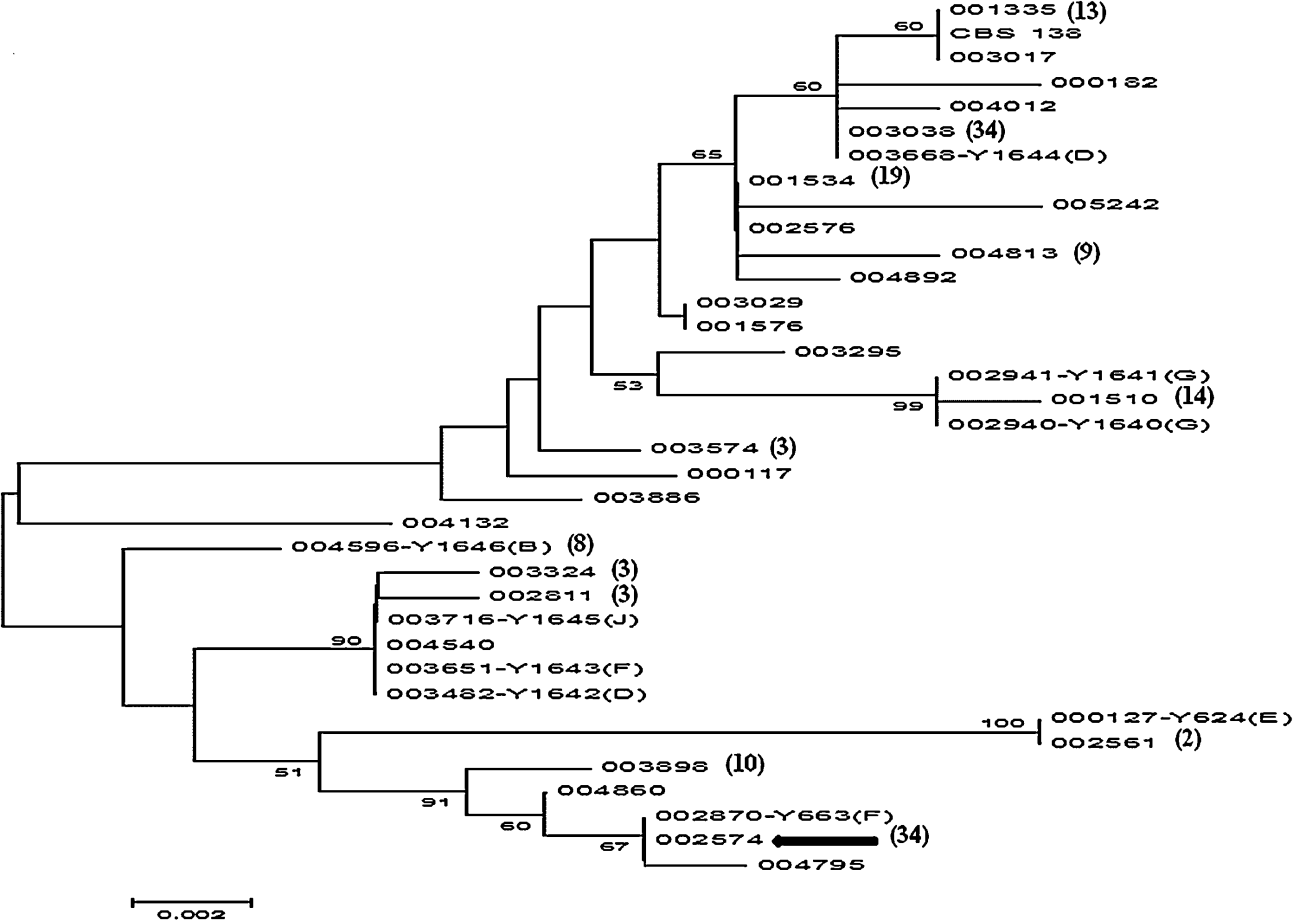
Phylogenetic relationship, as deduced by Neighbor-joining method, based on the IGS region located between the *CDH1* and *EPR6* genes. 35 (plus CBS 138) different haplotypes (representing isolate sequences which had the available 474 positions) were deduced. The strain numbers correspond to the museum numbers of the initial collection and can be found in Supplementary materials Table 1. The names of the strains with small chromosomes are followed by a *capital letter* pointing out which CBS 138 chromosome is related to the small chromosome. Among the analyzed strains several sequences belonged to the same haplotype. The appearance of each haplotype, in addition to the shown strain (and if different from 1), is written in the *brackets* following the strain/sequence designation. The strains belonging to each of these haplotypes can be found in Supplementary methods Table S1. The group 002574 (analyzed for their karyotypes in Fig. 3) is *arrowed*. The bootstrap values are shown on some branches and the tree is not rooted

In this study we analysed the identity and phylogenetic relationship of our clinical isolates through the sequencing of two genetic loci. Initially, we could see that ten strains from the original collection had quite a distinct D1/D2 domain (belonging to the nuclear 26S rDNA locus) polymorphism and apparently did not belong to *C. glabrata* based on the yeast species definition (Kurtzman 2006). Also the karyotypes of these strains were different from the *C. glabrata* ones (data not shown). Likely, these strains were misclassified during the initial determination and deposition and we later excluded them from further experiments and from the analysis shown in Fig. 1 and they are not shown in Supplementary materials Table S1. We obtained in total (including 40 previously determined ones, see Poláková et al. 2009) 192 sequences of the D1/D2 domain, and 192 sequences of IGS, mapping between the nuclear *CDH1* and *ERP6* genes, and these can be found in Supplementary materials Table S1. Seven different haplotypes of the D1/D2 locus, based on the analyzed 489 positions were obtained (Fig. 1). The difference between the CBS 138 sequence and the least related strain 003338 was observed at five positions (see also Fig. 1). According to the yeast species definition (Kurtzman 2006) this means that all strains belong to *C. glabrata*. When the fast evolving IGS locus was analyzed, a more pronounced polymorphism was detected (Fig. 2). Therefore, in Fig. 2 more distinctive sub-groupings than in Fig. 1 could be observed. Neighbor-joining and Maximum Parsimony methods defined the same small chromosome containing sub-groups (data not shown). In short, these experiments confirmed which of the strains in the collection were indeed *C. glabrata* and provided a basis to explain the origin of different molecular events (see later sections).

### Karyotypes

**Fig. 3:**
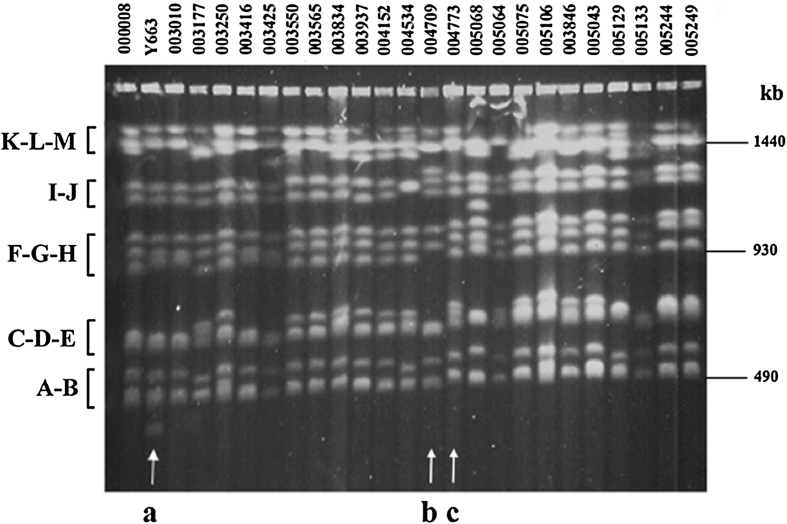
Electrophoretic karyotyping of 25 *C. glabrata* clinical isolates belonging to the same phylogenetic sub-group KA002574 which is *arrowed* in Fig. 2. Five groups of chromosomes (according to the CBS 138 nomenclature, see also Supplementary materials Fig. S1) are shown on the *left*, and the chromosome sizes on the right. The number of chromosome bands ranges from ten to thirteen but KA002870 (Y663) has fourteen chromosome bands because of its small chromosome (*arrowed* as a). The large chromosome group (K-L-M) shows a clear variation, from one band as in KA005064 to three bands, as in KA003250, or even four bands, as in KA005129. KA004709 and KA004773, *arrowed* as *b* and *c*, were isolated in 1997 from the same hospital but have clearly different karyotypes. In b we can see only ten bands but the third smallest chromosome (located in the C-D-E group) is likely a double band, while in *c* there are 12 bands

**Fig. 4:**
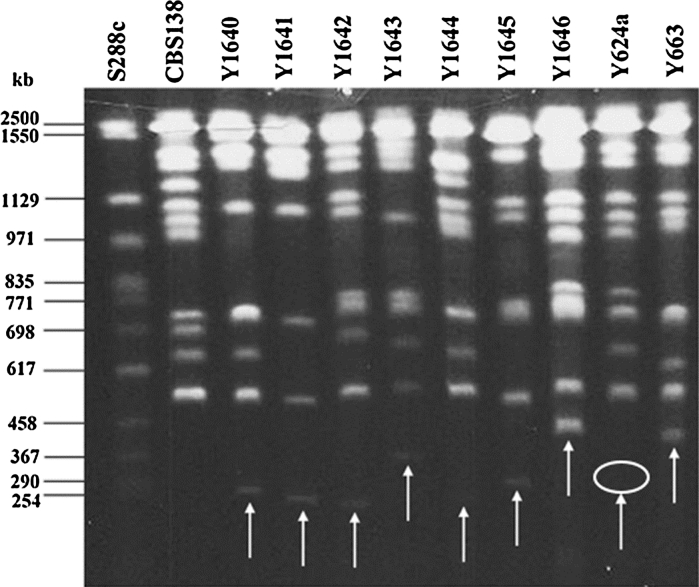
Electrophoretic karyotyping of nine clinical isolates of *C. glabrata* with small chromosomes. *S. cerevisiae* S288C (Y1307) and CBS 138 were included as references to determine the size of the new chromosomes. Y624a is a daughter strain of KA000127 (Y624) which has lost its small chromosome but the position of the small chromosome, as it would be in Y624, is *circled*. Y624 and its small chromosome were described previously (Poláková et al. 2009). The sizes of small chromosomes determined by calculation of chromosomal migration on the gel were estimated to be between 280 and 420 kb (see also Table 2). Note, strains Y1640 and Y1641 are from the same patient taken at different time points and the two small chromosomes have a slightly different size

The karyotypes of 151 isolates of *C. glabrata* were determined in this study, but in addition 40 strains had been analyzed already before (Poláková et al. 2009) thus providing 191 different karyotypes. There was apparent variation among the obtained karyotypes, ranging in the number of detected bands from ten to fourteen (Fig. 3). CBS 138 fourteen chromosomes are illustrated in Supplementary materials Fig. S1. The variation in the intensity of the chromosomal bands was also observed, and it could be explained as that some of the more intensive bands were composed of two or even more chromosomes. For example, Y663 has likely two double bands (of higher intensity then expected from the equal stoichiometric distribution), one in the K-L-M chromosome group and another in the C-D-E group. Figure 3 shows karyotypes originating from a set of strains belonging to the same phylogenetic sub-group KA002574 (see Fig. 2, the arrowed group). These strains are very closely related, but they exhibited a clear chromosomal polymorphism, in chromosome band numbers, sizes and intensity vary. Only one strain in this group, KA002870 (Y663) (Poláková et al. 2009), has 14 chromosome bands because of its small chromosome, while the remaining 24 strains show 10–13 bands (Fig. 3). The polymorphism is especially apparent within the large chromosomes K, L and M. This is in agreement with the previously published observations explaining the large chromosome polymorphism as a consequence of variation within the gene copy numbers at the rDNA locus (Muller et al. 2009). Among those 25 related isolates, we also found some that were isolated the same year from patients who were treated at the same hospital (Fig. 3; Supplementary material Table S1). However, even in these cases the karyotypes showed some degree of rearrangements. Interestingly, two strains from the same patient, KA002940 (Y1640) and KA002941 (Y1641), taken at different times of treatment, showed different karyotypes with 10–11 chromosomal bands detected (Fig. 4).

Among the 151 clinical isolates with newly determined karyotypes, we found seven strains, KA002940 (Y1640), KA002941 (Y1641), KA003482 (Y1642), KA003651 (Y1643), KA003668 (Y1644), KA003716 (Y1645), KA004596 (Y1646), having new small chromosomes within the size range of 280–420 kb (Table 1). These sizes are similar to those reported previously for the two small chromosomes belonging to KA000127 (Y624), and KA002870 (Y663) (Poláková et al. 2009). The nine strains with small chromosomes, seven new and two reported previously, are described in Table 1 and Fig. 4. We also tested all seven strains with small chromosomes for their resistance potential under in vitro conditions (Table 1).

**Table 1:** Characteristics of nine *Candida glabrata* clinical isolates with small chromosomes, the origin, size, stability of small chromosome and LD100 for fluconazole

Y number	Museum number	Source	Year of isolation	Hospital	Origin of small chromosome	Size of small chromosome (kb)	LD100 (μg/ml)	Copy number	Stability after 70 generations (%)
624Y	000127	Blood	1986	Braendstrup	E	330	80	2	60
663Y	002870	Faeces	1992	Rh5052^a^	F	390	1116.8	2	30
1640Y	002940	Blood	1993	Rh7806^b^	G	305	388.8	1	100
1641Y	002941	Blood	1993	Rh7806^b^	G	290	388.8	1	100
1642Y	003482	Blood	1994	Rh8223^c^	D	285	80	2	100
1643Y	003651	Unknown	1994	London	F	365	125	1	100
1644Y	003668	Blood	Before 1990	ATCC 90030	D	290	80	2	38
1645Y	003716	Blood	1994	Århus	J	332	1116.8	1	100
1646Y	004596	Blood	1997	Århus	B	420	1116.8	1	100

Note that Y1643 and Y1644 do not originate from Danish patients/hospitals. LD100 is the lethal dose (the lowest concentration which completely eliminates the growth of the yeast strain)

*ATCC* American type culture collection

^a^Rigshospitalet (Copenhagen), Department 5052

^b^Rigshospitalet (Copenhagen), Department 7806

^c^Rigshospitalet (Copenhagen), Department 8223

### Small chromosomes

It was assumed that each small chromosome contains one of the known centromeres. To investigate about the precise origin of the new small chromosomes, thirteen probes originating from genes in the vicinity of the CBS 138 centromeres were used in Southern analysis, and are listed in Supplementary materials, Table S2. In Fig. 2 each strain with a small chromosome also has a Y number in its designation and this is followed by a capital letter. These capital letters, B, D, E, F, G and J, indicate the relationship between the small chromosome and the CBS 138 chromosomes (Table 1). For example, in strain 002870 (Y663) F, the probe derived from a gene in the vicinity of the centromere from chromosome F, hybridized with the corresponding small chromosome. Two strains, 003482 and 003668, had their small chromosomes hybridized to the D chromosome probe, but they are not related and belong to two different strain sub-groups (Fig. 2). In addition, two strains, 002870 and 003651, had their small chromosomes hybridized to the F probe, and they are apparently not closely related (Fig. 2). Therefore, in both pairs of strains the corresponding small chromosomes originated independently from the parental D or F chromosome, respectively. On the other hand, the two strains (002940 and 002941) with their small chromosomes originating from chromosome G, are very closely related and they originate from the same patient (Fig. 2).

**Fig. 5:**
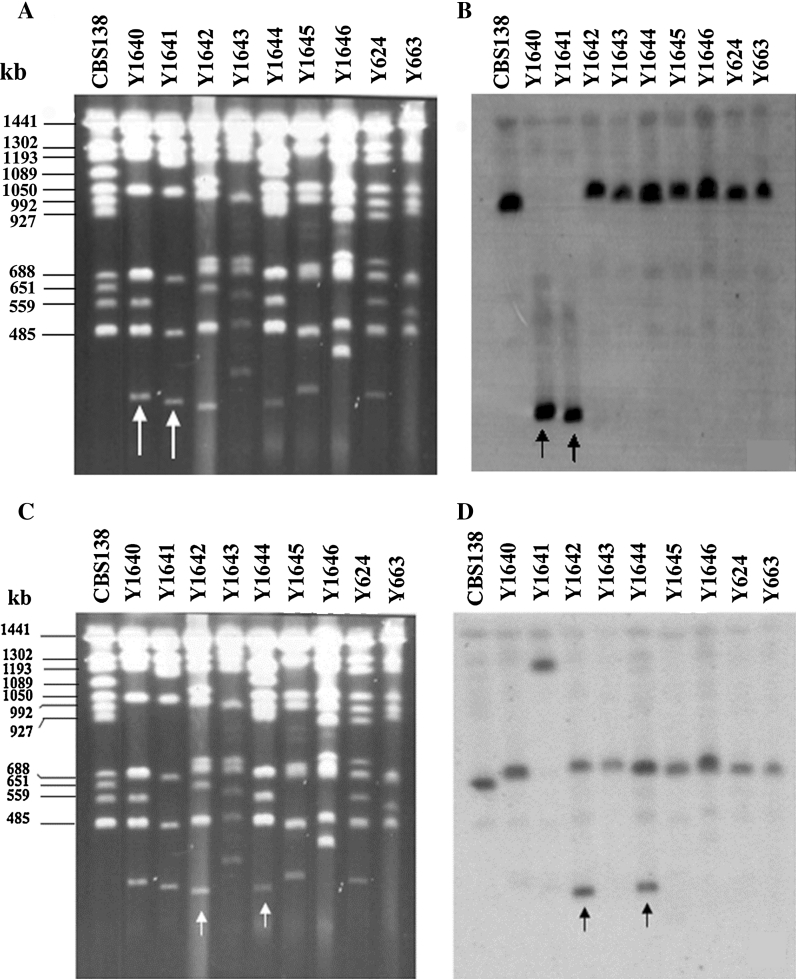
Chromosome separations (**a** and **c**) and Southern blots (**b** and **d**) of *C. glabrata* clinical isolates with small chromosomes. CBS 138 was used as a reference. The gel **a** was transferred to membrane **b**, which was hybridized with the “Gel” gene probe originating from CBS 138 chromosome G. The Y1640 and Y1641 small chromosomes (*arrowed*) hybridized to the gel probe showing that they share the origin with chromosome G. Note that in these two strains only one signal was obtained. The chromosomes from gel **c** were transferred to membrane **d** and hybridized with the probe “Dcl” (originating from chromosome D). Note that in both Y1642 and Y1644 there were two bands, the original chromosome and the small chromosome, hybridizing to the probe

In some cases the probe hybridized only to the small chromosome and not to the other chromosomes (Fig. 5; Supplementary material Figs. S2, S3, S4). For example, when we used the gene probe, called Gel, (Supplementary materials Table S2), on 002940 (Y1640) and 002941 (Y1641), we only obtained a signal from the small chromosome, and not from any larger chromosome (Fig. 5b). This could be, for example, explained by a translocation of the larger arm from the original chromosome G to another chromosome, while the left arm, the centromeric region and a part of the right arm remained as an autonomous, but smaller chromosome. However, also other mechanisms could additionally contribute to the origin of these chromosomes. While these two small chromosomes most likely have the same origin, the parental chromosome was upon the translocation event additionally remodeled giving two different sizes of 305 and 290 kb, respectively (Fig. 4).

Also the small chromosomes from Y1643, Y1645 and Y1646, having the size of 365, 332, 420 kb respectively, result from translocation events. For example, it seems possible that in Y1643 the right arm of chromosome F was translocated to another chromosome, leaving a 365 kb fragment (with the centromere) as a small chromosome. In Y1645 and Y1646 we could deduce rearrangements/translocations involving chromosomes J and B, respectively (Supplementary material Fig. S2 and S4). In the case of Y1642 and Y1644, the probe hybridizing to the small chromosome also hybridized to a larger chromosome D (Table 1; Fig. 5d). We explain these results as a partial duplication of chromosome D resulting in the 285 and 290 kb small chromosomes, respectively. The original centromere in these cases is present in two copies, on the parental and the small chromosome. Y1642 and Y1644, both carry a duplication of chromosome D, but the duplication had an independent origin (Fig. 2).

Three closely related strains, Y1642, Y1643 and Y1645, contain three different kinds of small chromosomes, originating from three different parental chromosomes (Fig. 2), and thus from independent events.

**Fig. 6:**
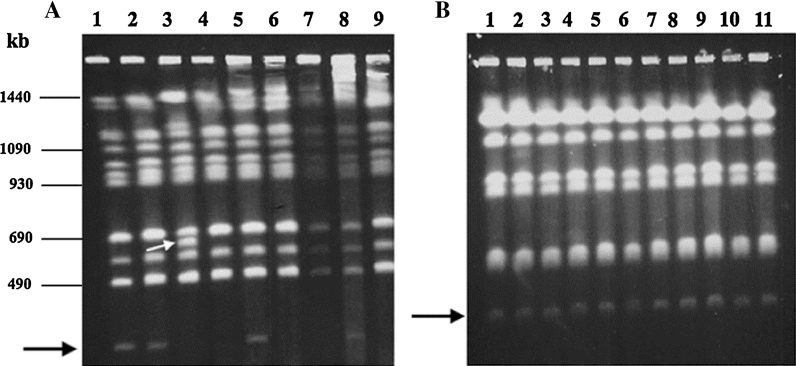
Chromosomal stability of two *C. glabrata* clinical isolates with small chromosomes grown in liquid YPD for 70 generations. **a** Karyotypes of the parental strain Y1644 (*lane 1*) and eight randomly selected progenies (*lanes 2*–*9*), the position of the small chromosome is indicated by a *black arrow*. Chromosome D rearrangement in one daughter lineage is *arrowed* in *white*. **b** Karyotypes of the parental strain Y1645 (*lane 1*) and ten (*lanes 2–11*) randomly selected progenies after 70 generations. The small chromosome is *arrowed*

A majority of the clinical isolates with these small chromosomes were stable for several generations when growing in a non-selective medium (YPD without fluconazole). As expected, Y1640, Y1641, Y1643, Y1645 and Y1646, were stable and retained their small chromosomes generated upon translocation, because a majority of genes located on the corresponding small chromosome were present in only one copy per genome. On the other hand, Y1644 was mitotically unstable and the small chromosome was lost in almost two thirds of the progeny and even chromosomal rearrangements could be observed in the resulting daughter lineages (Fig. 6a). The behavior of this strain was similar to the previously tested Y624 and Y663. The corresponding small chromosomes were a result of segmental duplications and therefore the small chromosome genes present in duplicate and thus the small chromosome could in principle be lost. In contrast, the small chromosome in Y1642 contains a partial duplication of chromosome D, and it was stable in our experiments and kept the novel small chromosome for 70 generations (Table 1). Y1642 was not particularly resistant to azole (Table 1) but the small chromosome could carry some single copy genes.

### Putative resistance genes on small chromosomes

In *C. glabrata*, several genes play a role in the interactions between the yeast and the host. It could be that some of the genes found on the small chromosomes are involved in the virulence and/or anti-fungal drug resistance of the strain. Thus, we examined all identified small chromosomes for the presence of any putative virulence and resistance genes. The region on left and right of the centromere, corresponding to the size of the small chromosome, was analyzed (Table 2), employing the published genome of the CBS 138 strain. Several resistance genes were found (Table 2).

**Table 2:** Analysis of newly characterized small chromosomes for the presence of the known virulence/resistance genes

Strain	Origin of small chromosomes	Genes	Presence of gene on the small chromosome^a^	Gene description	References
Y1642	D	CAGL0D03674g	–	ATP binding cassette family member	Vermitsky et al. (2006)
Y1644	D	CAGL0D03674g	–		
Y1640	G	CAGL0G00242g	+	ABC transporter, mediates export of organic anions including oligomycin, upregulated in azole-resistant strain	Tsai et al. (2010)
Y1641	G	CAGL0G00242g	+		
Y1643	F	*AUS1* (CAGL0F01419g)	+	ABC transporter involved in sterole uptake, azole resistance	Nakayama et al. (2007)
		*PDH1* (*CDR2*) (CAGL0F02717g)	+	ABC transporter involved in multidrug resistance	Tsai et al. (2006), Thakur et al. (2008), Vermitsky et al. (2006)
Y1645	J	CAGL0J00363g	+	Presumed antiporter of the *DHA1* family of multidrug resistance transporters	Vermitsky et al. (2006)
Y1646	B	CAGL0B02343g	+	Multidrug efflux pump of the major facilitator superfamily, required for resistance to aminotriazole and 4-nitroquinoline-*N*-oxide	Vermitsky et al. (2006) and Gbelska et al. (2006)

A putative gene was firstly predicted by bioinformatics tools and later confirmed by a Southern analysis

^a^The presence of the gene on the small chromosome was determined by Southern analysis

The duplicated segment of chromosome D, found in Y1642 and Y1644 small chromosomes, could encode CAGL0D03674g that is an ortholog of the *S. cerevisiae* *YPL226w* gene that might be involved in drug transport. This gene is highly similar to *C. albicans* *ELF1* conferring a drug-resistance phenotype (Sturtevant et al. 1998). However, our Southern analysis could not detect this gene on the small chromosome (Table 2, Supplementary materials Fig. S5A), and in addition, Y1642 and Y1644 are very sensitive on azole.

In both, Y1640 and Y1641, which are highly azole resistant, the region of 305 kb from the left end of chromosome G, which includes the centromere, also encodes the gene CAGL0G00242g, belonging to the ATP-binding cassette family and highly similar to the *S. cerevisiae* *YOR1* gene, which encodes an ABC transporter.

Y1643 carries a small chromosome which originates from chromosome F. Several genes from this part of chromosome F are known to be involved in the resistance potential of *C. glabrata*. For example, they encode a transporter of the ATP-binding cassette family, CAGL0F01419g, which is highly similar to the *S. cerevisiae* *AUS1* gene. In addition, the Y1643 small chromosome encodes the ATP-binding cassette family, CAGL0F02717, an ortholog of the *S. cerevisiae* ABC transporter *PDR5* gene (known as *PDH1* in *C. glabrata*), and involved in the transcriptional activation of pleiotropic drug resistance.

The small chromosome in Y1645 carries an ortholog of the *S. cerevisiae* *DHA1* family of multidrug resistance transporters (CAGL0J00363g) and this gene up-regulation results in reduced susceptibility to azoles.

In Y1646, 420 kb chromosome segments on both sides of the centromere of chromosome B carry CAGLA0B02343g that encodes a protein required for aminotriazole resistance, similar to *S. cerevisiae* *YML116* (*SNQ1*).

The strains with small chromosomes, which contained a putative resistance gene, were analyzed for the expression level of the corresponding six genes. The expression of four of these genes was not changed in the corresponding strain where the gene was located on the small chromosome (Supplementary materials Table S3). However, in the case of Y1646, which is highly resistant to azole, the expression of CAGLA0B02343g was more than two times elevated (Supplementary materials Table S3). This gene was highly expressed also in Y1642, which is not azole resistant.

### Generation of new chromosomes and conclusion

In this study we examined a unique collection of *C. glabrata* strains covering Danish hospitals during the period of 1985–1999. This time period is especially interesting because the main anti-fungal agents used nowadays (based on azoles), were introduced to Denmark in early 90s. Only limited sequence variability was detected in the D1/D2 domain (Fig. 1). However, when a fast evolving locus, covering the intergenic region between two ORFs, was examined (Fig. 2), several phylogenetic sub-groups were found. Even strains with a very similar intergenic locus sequence, belonging to the same phylogenetic sub-group, had variable karyotypes (e.g. Fig. 3; Supplementary materials Fig. S6), confirming the previous suggestion that the *C. glabrata* chromosomes rearrange faster than point mutations accumulate within the genome sequence (Poláková et al. 2009). One could speculate that each patient evolved its own non-pathogenic strain into a virulent one, able to cause a systemic infection under immuno-suppressed conditions.

Nine strains with small chromosomes (Fig. 4) belong to different sub-clades (Fig. 2), Y1642, Y1643 and Y1645 belong to a closely related group of strains (sub-group KA004540, in Fig. 2) and this clade gave rise to three different type of small chromosomes, related to the CBS 138 chromosome D, F and J, respectively. Apparently, the common progenitor of these strains was very prone to generate small chromosomes. D and F related small chromosomes appear also in distant clusters. In this report, we describe a new mechanism for generation of small chromosomes, through chromosomal breakage and translocation of a centromere-less arm to another chromosome (Fig. 5; Supplementary material Figs. S2, S3, S4). Such translocations could be reciprocal or non-reciprocal, and are stable because the cell cannot tolerate a loss of the small chromosome (Table 1). While segmental duplications increase the gene dosage, the translocation pathway does not. When we examined the putative resistance gene CAGL0B02343g in the strain Y1646 (Table 2), we could see that the expression was significantly elevated (Supplementary material Table S3). One could then speculate that the high azole resistance phenotype of this strain (Table 1) is somehow connected with the over-expression of the CAGL0B02343g gene coding for a multi-drug efflux pump. However, this gene is also highly expressed in Y1642, which is not very resistant on azole. We conclude that the small chromosomes contain more than the here traced genes and it appears likely that some of these may contribute to an enhanced propagation in the patient.

It seems that in our collection approximately each twentieth strain employed a strategy of the small chromosome generation (Table 1). In addition, it could also be that some strains had lost their small chromosome during the preservation and growth under non-selective conditions in the laboratory medium. Generation of a new chromosome can provide genome configurations which could be more competitive, for example by increasing the anti-fungal resistance in a certain patient habitat, and thus successfully proliferate in a relatively hostile niche. While we described two paths of small chromosome generation, additional mechanisms may have been involved in the generation of the observed rearrangements.

## Electronic supplementary material

Below is the link to the electronic supplementary material.

Supplementary material 1 (DOCX 6954 kb)
