# Supplementary material for: Small chromosomes among Danish Candida glabrata isolates originated through different mechanisms
Source: Antonie Van Leeuwenhoek. 2013 May 14;104(1):111–22. doi: 10.1007/s10482-013-9931-3 (PMC3672514; doi:10.1007/s10482-013-9931-3)
Supplement: Supplementary file 1 — Supplementary material 1 (DOCX 6954 kb) [file 10482_2013_9931_MOESM1_ESM.docx]

**Supplementary materials**

**for**

**Small chromosomes among Danish *Candida glabrata* isolates originated through different mechanisms**

Khadija Mohamed Ahmad^1^, Olena P. Ishchuk^1^, Linda Hellborg^1^, Gloria Jørgensen^2^, Miha Škvarč^1^, Jørgen Stenderup^3^, Dorte Jørck-Ramberg^2^, Silvia Polakova^1^ and Jure Piškur^1^

^1^ Department of Biology, Lund University, Sölvegatan 35, Lund SE-223 62, Sweden, e-mail: [Khadija_Mohamed.Ahmad@biol.lu.se](mailto:Khadija_Mohamed.Ahmad@biol.lu.se)

^2^ BioCentrum-DTU, Technical University of Denmark, DK-2800 Lyngby, Denmark

^3^Department of Clinical Microbiology, Regionshospitalet Herning, DK-7400 Herning, Denmark

**Table S1**. 192 clinical isolates of *Candida glabrata* collected from Danish patients and now also preserved at the Lund University. The museum numbers, KA, refer to the initial collection at the Danish “Statens Serum Institute”. The table shows the year of origin, the hospital, the source of isolation of the sample, fluconazole sensitivity and Gene Bank accession numbers for the two sequenced loci.

| **IGS (CDH1-ERP6) GenBank Acc.no.** | **LD100 (µg/ml)** | **LSU rDNA Gene bank Acc. no.** | **Source of isolate** | **Place of Isolation** | **Year of isolation** | **Museum Number** |
| --- | --- | --- | --- | --- | --- | --- |
| HM591876 | 1116.8 | HM591699 | Urine | Kommunehosp | 1985 | KA000008 |
| HM591877 | 125 | HM591700 | Blood | Bispebjerg hospital | 1985 | KA000012 |
| HM591905- *AM982691 | 80 | AF369491 | Unknown | Rh7806 ** | 1986 | KA000117 |
| HM591906- *AM982692 | 45 | AF369492 | Blood | Roskilde | 1986 | KA000119 |
| HM591907- *AM982693 | 80 | AF369493 | Blood | Brandstrup | 1986 | KA000127  (Y 624) |
| HM591908- *AM982694 | 45 | AF369494 | Lymph node | Rh7806** | 1986 | KA000149 |
| HM591909- *AM982695 | 45 | AF369495 | Palate | Herlev | 1986 | KA000179 |
| HM591913- *AM982696 | 125 | AF369496 | Blood | Unknown | 1986 | KA000182 |
| HM591878 | 1116.8 | HM591701 | Faeces | Finseninst | 1986 | KA000291 |
| HM591879 | 388.8 | HM591702 | Sputum | Gentofte | 1986 | KA000300 |
| HM591880 | 45 | HM591703 | Urine | Hvidovre | 1987 | KA000320 |
| HM591881 | 45 | HM591704 | Sputum | Rh7809** | 1987 | KA000365 |
| HM591882 | 80 | HM591705 | Suprarenal gland | Rh7703** | 1987 | KA000372 |
| n.d. | 45 | HM591706 | Neck | Unknown | 1987 | KA000540 |
| HM591883 | 45 | HM591707 | Faeces | Rh7806** | 1987 | KA000571 |
| HM591914 | 45 | HM591708 | Urine | Rh7806** | 1988 | KA000729 |
| HM591884 | 45 | HM591709 | Throat | Rh7806** | 1988 | KA000732 |
| HM591885 | 45 | HM591710 | Unknown | Hvidovre | 1988 | KA000737 |
| HM591886 | 45 | HM591711 | Blood | Hvidovre | 1988 | KA000801 |
| HM591887 | 45 | HM591712 | Blood | Bispebjerg hospital | 1988 | KA000870 |
| HM591915 | 1116.8 | HM591713 | Blood | Rh8223** | 1988 | KA000892 |
| HM591888 | 80 | HM591714 | Blood | Nykobing | 1989 | KA001145 |
| HM591889 | 15 | HM591715 | Vagina | Nykobing.F | 1989 | KA001207 |
| HM591890 | 45 | HM591716 | Blood | Århus | 1989 | KA001211 |
| HM591891 | 1116.8 | HM591717 | Blood | Roskilde | 1989 | KA001224 |
| HM591899 | 45 | HM591721 | Blood | Herlev | 1989 | KA001334 |
| HM591900 | 45 | HM591722 | Blood | Herlev | 1989 | KA001335 |
| n.d. | 45 | HM591723 | Drop | Alborg | 1990 | KA001457 |
| HM591910 | 388.8 | HM591724 | Blood | Arhus | 1990 | KA001510 |
| HM591911 | 45 | HM591725 | Blood | Naestved | 1990 | KA001528 |
| HM591912 | 45 | HM591726 | Blood | Roskilde | 1990 | KA001534 |
| HM591901 | 45 | HM591727 | Blood | Roskilde | 1990 | KA001535 |
| HM591917 | 1116.8 | HM591728 | Blood | Arhus | 1990 | KA001576 |
| HM591902 | 45 | HM591729 | Sputum | Rh7806** | 1990 | KA001687 |
| HM591903 | 1116.8 | HM591730 | Unknown | Arhus | 1990 | KA001713 |
| HM591904 | 15 | HM591731 | Unknown | Rh5101** | 1991 | KA001740 |
| HM591732- *AM982698 | 388.8 | HM591612- *AF369498 | Unknown | London 2514 | 1992 | KA002516 |
| HM591733 | 80 | HM591613 | Unknown | Rh5101** | 1992 | KA002553 |
| HM591858- *AM982699 | 45 | HM591614- *AF369500 | Faeces | Rh7806** | 1992 | KA002561 |
| HM591809- *AM982700 | 45 | HM591638- *AF369501 | Throat | Rh5101** | 1992 | KA002574 |
| HM591763- *AM982701 | 15 | HM591540- *AF369502 | Unknown | Rh5101** | 1992 | KA002576 |
| HM591764- *AM982702 | 388.8 | HM591615- *AF369503 | Gland | Gentofte | 1992 | KA002684 |
| HM591810- *AM982704 | 1116.8 | HM591640- *AF369504 | Blood | Viborg | 1992 | KA002706 |
| HM591786 | 45 | HM591561 | Unknown | Rh5101** | 1992 | KA002718 |
| HM591765- *AM982705 | 45 | HM591557- *AF369505 | Catheter spit | Hvidovre | 1992 | KA002736 |
| HM591916- *AM982706 | 388.8 | HM591539- *AF369506 | Blood | Slagelse | 1992 | KA002742 |
| HM591859- *AM982707 | 80 | HM591569- *AF369507 | Faeces | Copenhagen-Rigs hospital | 1992 | KA002792 |
| HM591837 | 45 | HM591570 | Blood | Hillerod | 1992 | KA002797 |
| HM591766- *AM982708 | 15 | HM591542- *AF369509 | Blood | Bispebjerg hospital | 1992 | KA002806 |
| HM591734- *AM982709 | 45 | HM591543- *AF369510 | Blood | Bispebjerg hospital | 1992 | KA002807 |
| HM591735- *AM982710 | 45 | HM591616- *AF369511 | Blood | Bispebjerg hospital | 1992 | KA002808 |
| HM591787- *AM982711 | 45 | HM591641- *AF369512 | Faeces | Hillerod | 1992 | KA002811 |
| HM591918 | 45 | HM591642 | Oesophagus | Ålborg | 1992 | KA002860 |
| HM591860 | 45 | HM591607 | Sputum | Holstebro | 1992 | KA002861 |
| HM591736- *AM982713 | 15 | HM591617- AF369516 | Blood | Koge | 1992 | KA002865 |
| HM591788-*AM982714 | 15 | HM591562- *AF369517 | Blood | Koge | 1992 | KA002866 |
| HM591767- *AM982715 | 15 | HM591544- *AF369518 | Blood | Koge | 1992 | KA002867 |
| HM591768- *AM982716 | 1116.8 | HM591545- *AF369519 | Faeces | Rh5052** | 1992 | KA002870  (Y663) |
| HM591737- *AM982718 | 388.8 | HM591618- *AF369521 | Faeces | Unknown | 1992 | KA002895 |
| HM591769- *AM982720 | 45 | HM591600- *AF369523 | Blood | Århus | 1992 | KA002901 |
| HM591770 | 15 | HM591601 | Faeces | Rh5062** | 1993 | KA002914 |
| HM591738 | 15 | HM591602 | Unknown | Rh5101** | 1993 | KA002924 |
| HM591919 | 45 | HM591676 | Faeces | Rh2143** | 1993 | KA002927 |
| HM591789 | 388.8 | HM591644 | Blood | Rh7806** | 1993 | KA002940  (Y1640) |
| HM591790 | 388.8 | n.d. | Blood | Rh7806** | 1993 | KA002941  (Y1641) |
| HM591791 | 125 | HM591571 | Faeces | Hvidovre | 1993 | KA002960 |
| HM591739 | 125 | HM591677 | Blood | Hvidovre | 1993 | KA002992 |
| HM591771 | 15 | HM591603 | Faeces | Unknown | 1993 | KA002994 |
| HM591740 | 1116.8 | HM591619 | Faeces | Rh8223** | 1993 | KA003010 |
| HM591792 | 15 | HM591572 | Faeces | Rh5062** | 1993 | KA003017 |
| HM591793 | 1116.8 | HM591645 | Blood | Ålborg | 1993 | KA003029 |
| HM591838 | 15 | HM591573 | Blood | Rh8223** | 1993 | KA003038 |
| HM591741 | 45 | HM591620 | Blood | Rh8223** | 1993 | KA003067 |
| HM591794 | 15 | HM591564 | Unknown | Hvidovre | 1993 | KA003085 |
| HM591742 | 1116.8 | HM591604 | Unknown | Hvidovre | 1993 | KA003136 |
| HM591743 | 80 | HM591605 | Tracheal | Bispebjerg hospital | 1993 | KA003153 |
| HM591744 | 45 | HM591621 | Faeces | Århus | 1993 | KA003177 |
| HM591795 | 125 | HM591574 | Sputum | Farso | 1993 | KA003197 |
| HM591839 | 1116.8 | HM591622 | Faeces | Rh4041** | 1993 | KA003215 |
| HM591745 | 15 | HM591606 | Faeces | Roskilde | 1993 | KA003250 |
| HM591746 | 80 | HM591608 | Nose | Hvidovre | 1993 | KA003251 |
| HM591772 | 15 | HM591546 | Blood | Århus | 1993 | KA003295 |
| HM591747 | 45 | HM591623 | Blood | Nykobing F | 1993 | KA003310 |
| HM591748 | 45 | HM591609 | Blood | Rh8223** | 1993 | KA003324 |
| HM591773 | 45 | HM591624 | Blood | Rh7806** | 1993 | KA003325 |
| HM591811 | 45 | HM591575 | Unknown | Århus | 1993 | KA003338 |
| HM591749 | 80 | HM591678 | Blood | ATCC 90030 | Before 1990 | KA003344 |
| HM591750 | 15 | HM591610 | Blood | Bispebjerg hospital | 1993 | KA003379 |
| HM591840 | 1116.8 | HM591550 | Pulmonary aspiration | Rh8223** | 1994 | KA003416 |
| HM591751 | 80 | HM591611 | Faeces | Århus | 1994 | KA003418 |
| HM591796 | 125 | HM591646 | Pulmonary aspirate | Rh8223** | 1994 | KA003425 |
| HM591841 | 45 | HM591551 | Urine | Hillerod | 1994 | KA003464 |
| HM591774 | 15 | HM591565 | Urine | Randers | 1994 | KA003481 |
| HM591775 | 80 | HM591566 | Blood | Rh8223** | 1994 | KA003482  (Y1642) |
| HM591776 | 125 | HM591576 | Blood | Århus | 1994 | KA003496 |
| HM591812 | 45 | HM591647 | Blood | Århus | 1994 | KA003501 |
| HM591842 | 45 | HM591568 | Blood | Randers | 1994 | KA003503 |
| HM591843 | 45 | HM591680 | Mouth | Bispebjerg hospital | 1994 | KA003538 |
| HM591844 | 15 | HM591547 | Blood | Rh9301** | 1994 | KA003550 |
| HM591825 | n.d. | HM591664 | Blood | Rh9301** | 1994 | KA003559 |
| HM591845 | 15 | HM591625 | Blood | Rh9301** | 1994 | KA003565 |
| HM591752 | 15 | HM591552 | Blood | Århus | 1994 | KA003574 |
| HM591778 | 125 | HM591577 | Blood | Århus | 1994 | KA003601 |
| HM591753 | 125 | HM591626 | Unknown | (London) | 1994 | KA003651  (Y1643) |
| HM591777 | 80 | HM591578 | Blood | ATCC90030 | Before 1990 | KA003668  (Y1644) |
| HM591754 | 80 | HM591627 | Blood | Odder | 1994 | KA003683 |
| HM591846 | 45 | HM591628 | Sputum | Rh7806** | 1994 | KA003694 |
| HM591755 | 80 | HM591629 | Sputum | Rh7806** | 1994 | KA003696 |
| HM591756 | 1116.8 | HM591630 | Blood | Århus | 1994 | KA003716  (Y1645) |
| HM591779 | 125 | HM591591 | Blood | Rh9301** | 1994 | KA003726 |
| HM591813 | 45 | HM591592 | Blood | Rh9301** | 1994 | KA003739 |
| HM591814 | 15 | HM591593 | Blood | Esbjerg | 1995 | KA003777 |
| n.d. | 45 | HM591594 | Blood | Esbjerg | 1995 | KA003781 |
| HM591797 | 45 | HM591681 | Blood | Bispebjerg hospital | 1995 | KA003786 |
| HM591815 | 1116.8 | HM591579 | Trachea | Hvidovre | 1995 | KA003821 |
| HM591816 | 1116.8 | HM591580 | Faeces | Århus | 1995 | KA003834 |
| HM591826 | 125 | HM591665 | Faeces | Århus | 1995 | KA003846 |
| HM591847 | 45 | HM591553 | Saliva | Rh9301** | 1995 | KA003867 |
| HM591798 | 15 | HM591648 | Blood | Esbjerg | 1995 | KA003886 |
| HM591817 | 1116.8 | HM591595 | Blood | Århus | 1995 | KA003890 |
| HM591818 | 15 | HM591596 | Blood | Rh9301** | 1995 | KA003898 |
| HM591827 | 45 | HM591666 | Faeces | Nykobing F | 1995 | KA003901 |
| HM591799 | 45 | HM591649 | Blood | Nykobing F | 1995 | KA003904 |
| HM591848 | 1116.8 | HM591631 | Blood | Esbjerg | 1995 | KA003937 |
| HM591819 | 15 | HM591650 | Blood | Esbjerg | 1995 | KA003943 |
| HM591898 | 15 | HM591597 | Blood | Rh8706** | 1995 | KA003947 |
| HM591757 | 45 | HM591632 | Abscess | Rh9301** | 1995 | KA004006 |
| HM591828 | 45 | HM591667 | Blood | Fr.berg | 1995 | KA004012 |
| HM591829 | 15 | HM591668 | Faeces | Rh2042** | 1995 | KA004015 |
| HM591849- *AM982684 | 15 | HM591581- *AF369484 | Blood | Rh9301** | 1996 | KA004114 |
| HM591830- *AM982685 | 1116.8 | HM591669- *AF369485 | Blood | Nykobing F | 1996 | KA004132 |
| HM591820 | 125 | HM591651 | Blood | Rh9301** | 1996 | KA004143 |
| HM591758 | 45 | HM591633 | CVK | Odense | 1996 | KA004152 |
| HM591896 | 45 | HM591598 | Unknown | Bispebjerg hospital | 1996 | KA004161 |
| HM591897- *AM982686 | 15 | HM591599- *AF369486 | Unknown | Århus | 1996 | KA004492 |
| HM591831 | 125 | HM591670 | Blood | Viborg | 1996 | KA004509 |
| HM591780- *AM982688 | 15 | HM591582- *AF369488 | Blood | Bispebjerg hospital | 1996 | KA004524 |
| HM591832- *AM982689 | 45 | HM591671-  *AF369489 | Blood | Bispebjerg hospital | 1996 | KA004534 |
| HM591850-  *AM982690 | 45 | HM591554-  *AF369490 | Blood | Ålborg | 1996 | KA004540 |
| HM591759 | 1116.8 | HM591634 | Blood | Århus | 1997 | KA004596  (Y1646) |
| HM591800 | 15 | HM591652 | Dialysis | Herning | 1997 | KA004651 |
| HM591833 | 45 | HM591672 | Blood | Bispebjerg hospital | 1997 | KA004671 |
| HM591851 | 15 | HM591635 | Blood | Århus | 1997 | KA004709 |
| HM591781 | 388.8 | HM591583 | Oesophagus | Nykobing F | 1997 | KA004736 |
| HM591834 | 15 | HM591673 | Blood | Ålborg | 1997 | KA004751 |
| HM591760 | 1116.8 | HM591555 | Blood | Århus | 1997 | KA004773 |
| HM591852 | 388.8 | HM591556 | Blood | Hvidovre | 1997 | KA004775 |
| HM591801 | 125 | HM591682 | Tooth | Herlev | 1997 | KA004781 |
| HM591853 | 1116.8 | HM591548 | Urine | Viborg | 1997 | KA004795 |
| HM591802 | 45 | HM591653 | Blood | Hillerod | 1997 | KA004807 |
| HM591803 | 45 | HM591654 | Blood | Herlev | 1997 | KA004809 |
| HM591782 | 80 | HM591584 | Drain | Århus | 1997 | KA004813 |
| HM591854 | 45 | HM591636 | Breast | Bispebjerg hospital | 1997 | KA004823 |
| HM591783 | 15 | HM591567 | Blood | Århus | 1997 | KA004826 |
| HM591821 | 45 | HM591655 | Unknown | Herlev | 1997 | KA004858 |
| HM591923 | 80 | HM591549 | Unknown | Herlev | 1997 | KA004860 |
| HM591804 | 45 | HM591656 | Blood | Naestved | 1997 | KA004867 |
| HM591835 | 45 | HM591674 | Blood | Roskilde | 1997 | KA004869 |
| HM591855 | 45 | HM591585 | Autopsy | Retsmed | 1997 | KA004878 |
| HM591784 | 388.8 | HM591586 | Blood | Rh9301** | 1997 | KA004892 |
| HM591761 | 1116.8 | HM591637 | Blood | Statens Serum Institute | 1997 | KA004903 |
| HM591762 | 1116.8 | HM591558 | Blood | Århus | 1998 | KA004923 |
| HM591805 | 45 | HM591658 | Blood | Bispebjerg | 1998 | KA004931 |
| HM591806 | 45 | HM591659 | Blood | Herning | 1998 | KA004943 |
| HM591836 | 15 | HM591675 | Sputum | Vejle | 1998 | KA004951 |
| HM591807 | 15 | HM591660 | Blood | Århus | 1998 | KA005011 |
| HM591925 | 15 | HM591587 | Kidney | Slagelse | 1998 | KA005030 |
| HM591785 | 45 | HM591588 | Blood | Odense | 1998 | KA005033 |
| HM591856 | 45 | HM591559 | Blood | Nykobing F | 1998 | KA005041 |
| HM591808 | 45 | HM591661 | Blood | Hvidore | 1998 | KA005043 |
| HM591822 | 45 | HM591560 | Peritoneum | Ålborg | 1998 | KA005064 |
| HM591823 | 45 | HM591662 | Unknown | Bispebjerg hospital | 1998 | KA005068 |
| HM591824 | n.d. | HM591663 | Blood | Nykobing F | 1998 | KA005075 |
| HM591926 | 15 | HM591589 | Unknown | London 5097 | 1998 | KA005094 |
| HM591857 | 15 | HM591590 | Trachea | Statens Serum Institute | 1998 | KA005097 |
| HM591861 | 15 | HM591685 | Blood | Ålborg | 1998 | KA005103 |
| HM591862 | 1116.8 | HM591686 | Blood | Hilleod | 1998 | KA005105 |
| HM591863 | n.d. | HM591687 | Blood | Hvidore | 1998 | KA005106 |
| HM591864 | 45 | HM591688 | Blood | Ålborg | 1998 | KA005111 |
| HM591865 | 80 | HM591689 | Blood | Århus | 1998 | KA005118 |
| HM591866 | 80 | HM591690 | Blood | Hilleod | 1998 | KA005129 |
| HM591867 | 45 | HM591691 | Blood | Århus | 1998 | KA005133 |
| HM591927 | 45 | HM591719 | Blood | Ålborg | 1998 | KA005146 |
| HM591868 | 15 | HM591692 | Blood | Ålborg | 1998 | KA005159 |
| HM591869 | 388.8 | HM591693 | Blood | Århus | 1998 | KA005160 |
| HM591871 | 125 | HM591695 | Blood | Ålborg | 1999 | KA005229 |
| HM591872 | 15 | HM591696 | Blood | Odense | 1999 | KA005242 |
| HM591873 | 15 | HM591697 | Blood | Århus | 1999 | KA005243 |
| HM591874 | 45 | n.d. | Blood | Statens Serum Institute | 1999 | KA005244 |
| HM591875 | 80 | HM591698 | Blood | Naestved | 1999 | KA005247 |
| HM591894 | 80 | HM591720 | Blood | Alborg | 1999 | KA005249 |
| HM591895  HM591895 | 45 | n.d. | Blood | Herning | 1999 | KA005255 |

^a^ n.d. means not determined

^b^* means the sequence has two accession numbers in GenBank because the strain has been analyzed previously (in an independent study)

^c^ ** Rh means Rigshospitalet (Copenhagen) and usually the Department number is shown.

^d^ CVK (Central venous catheter).

^e^LD100 is the lethal dose of azole (the lowest concentration which completely eliminates the growth of the yeast)

**The strains belonging to the same haplotype groups, as presented in Fig. 1 and Fig. 2 of the main text:**

**Regarding Figure 1**

The following strains belong to the same D1/D2 haplotype as CBS 138:

000008, 000012, 000291, 000300, 000320, 000365, 000372, 000540, 000571, 000732, 000737, 000801, 000870, 000892, 001145, 001211, 001224, 001334, 001335, 001457, 001510, 001528, 001534, 001535, 001576, 001687, 001713, 001740, 002516, 002553, 002561, 002574, 002576, 002684, 002706, 002718, 002736, 002742, 002792, 002797, 002806, 002807, 002808, 002811, 002860, 002861, 002865, 002866, 002867, 002870, 002895, 002901, 002914, 002924, 002927, 002940, 002960, 002992, 002994, 003010, 003017, 003029, 003038, 003067, 003085, 003136, 003153, 003177, 003197, 003215, 003250, 003251, 003295, 003310, 003324, 003325, 003344, 003379, 003416, 003418, 003425, 003464, 003481, 003482, 003496, 003501, 003503, 003538, 003550, 003559, 003565, 003574, 003601, 003651, 003668, 003683, 003694, 003696, 003716, 003726, 003739, 003777, 003786, 003821, 003834, 003846, 003867, 003886, 003890, 003898, 003901, 003904, 003937, 003943, 003947, 004006, 004012, 004015, 004114, 004132, 004143, 004152, 004161, 004492, 004509, 004524, 004534, 004540, 004596, 004651, 004671, 004709, 004736, 004751, 004773, 004775, 004781, 004795, 004807, 004809, 004813, 004823, 004826, 004858, 004860, 004867, 004869, 004878, 004892, 004923, 004931, 004943, 004951, 005011, 005030, 005033, 005041, 005043, 005064, 005068, 005075, 005094, 005097, 005103, 005105, 005106, 005111, 005118, 005129, 005133, 005146, 005159, 005160, 005229, 005242, 005243, 005249.

**Regarding Figure 2**

The following strains belong to different haplotype groups based on the IGS region:

**001335** (000179, 000732, 001334, 002516, 002684, 003338, 003481, 003559, 003726, 004736, 004867, 004869, 005011)

**001510** (000012, 000737, 000870, 001224, 002736, 002742, 002960, 003067, 003197, 003890, 004143, 004781, 005030, 005146)

**001534** (000729, 001535, 001687, 001713, 001740, 002553, 002865, 002867, 002924, 002992, 003153, 003418, 003694, 003739, 003901, 004161, 004807, 004903, 005229)

**002561** (003215, 003601)

**002574** (000008, 000119, 000372, 001528, 002797, 002895, 003010, 003177, 003250, 003416, 003425, 003550, 003565, 003834, 003846, 003937, 004152, 004534, 004709, 004773, 004775, 004878, 005043, 005064, 005068, 005075, 005097, 005105, 005106, 005118, 005129, 005133, 005244, 005249)

**002811** (000365, 002792, 005103)

**003038** (000320, 000571, 000801, 000892, 001145, 001207, 001211, 002718, 002861, 002866, 002901, 002914, 002927, 003251, 003344, 003310, 003496, 003501, 003503, 003538, 003777, 003867, 003904, 004671, 004809, 004858, 004943, 004951, 005041, 005094, 005111, 005159, 005160, 005247)

**003324 (**003683, 003821, 004923)

**003574** (000291, 003464, 004492)

**003898** (000149, 003085, 003947, 004015, 004114, 004524, 004751, 004823, 004931, 005243)

**004596** (000300, 002806, 002807, 002808, 003325, 003379, 004006, 004509)

**004813** (002860, 002994, 003136, 003786, 003943, 004651, 004826, 005033, 005255)

**Table S2.** PCR primers used to prepare gene probes for Southern blot analysis of yeast chromosomes to determine the origin of the small chromosomes. The target genes covered by these primers, map close to the centromere regions of CBS 138. Also primers used to detect the presence of putative resistance genes are shown.

| **Hybridization Probe name** | **Primer name** | **Primer(5`-3`)** | **Product size, bp** | **Chromosomal position, bp** |
| --- | --- | --- | --- | --- |
| Acr | A00896g for | GGCAACTTGGCGTACTCTTAGATTC | 993 | A, 87887-88879 |
|  | A00896g rev | GCAGGTAATTCACAACGCAGGTC |  |  |
| Bcl | B04631g for | GACTGGTAACAAAGGTGCTGTCTC | 989 | B, 447475-448463 |
|  | B04631g rev | CTTGTGGACTCAAGACAGTTGGC |  |  |
| Cm2 | C04048g for | GTACTGTGAATGTGGCCGATACC | 1080 | C,400411-401490 |
|  | C04048g rev | GTCTGTAGCTCAAGGTTGTGCC |  |  |
| Dcl | D05808g for | CGCACAATGTCACACAGATGTGG | 1083 | D, 553003-554086 |
|  | D05808g rev | CGGTCAGCTTAGGATGGTTTGC |  |  |
| Plb3 | E02321g for | GCAGGTATACTGTCTGCATTCGAC | 1028 | E, 222799-223826 |
|  | E02321g rev | GAAGATCCAATCAGGCCATTGCAG |  |  |
| Fm2 | F02541g for | GCCTACTTCATCTTACGACTCAAGC | 1079 | F, 248382-249460 |
|  | F02541g rev | GATCACCAGATGTATCTTCACCAGC |  |  |
| Gel | G00374g for | GGCAGACTTGTCACCAACAAAGAG | 1067 | G, 37451-38517 |
|  | G00374g rev | CAATAGGAGCCTCTGGGATGACTAAC |  |  |
| Hcl | H09746g for | GCACAGCTAGTTAATGCACTTCCAG | 988 | H, 952005-952992 |
|  | H09746g rev | CTCTGAATGCTCTACCGTTTGGAAG |  |  |
| Im2 | I07821g for | GCAATTGGACAATCTGTCGAACACG | 985 | I, 753245-754229 |
|  | I07821g rev | GGAGACTGTACACCAGTGAGATAGG |  |  |
| Jm2 | J02530g for | GTGCTTTGGCGATGATAAGACCAG | 975 | J, 248631-249605 |
|  | J02530g rev | CTTGGCCTTGTTCACCTTGTCC |  |  |
| Kcr | K02937g for | CAGTAACTGCAGCAGTGACTTCAC | 1005 | K, 260558-261561 |
|  | K02937g rev | TTGATCTGGCATACCAATTCTGGTC |  |  |
| Lcr | L09130g for | GCACATTCCCTCAGAATCCAGC | 1018 | L, 990366-991383 |
|  | L09130g rev | GCGTCATCTATCAGTCCAGCTC |  |  |
| Mcr | M10527g for | CAAGGTGCCGTGAAATGGCTAC | 1026 | M,1052528-1053553 |
|  | M10527g rev | CAACGTCAGGAGCAGTAACAGC |  |  |
| CAGL0D03674g | KA1 for | CCCTGTCGACGCTCTGACCTCC | 859bp | D, 370821- 371679 |
|  | KA2 rev | GACGACGGCTTCGTTATCAGCACC |  |  |
| CAGL0G00242g | KA3 for | GGAGGGCGACGAATATGAGGTTACTG | 865bp | G, 21502-22366 |
|  | KA4 rev | GGCAACCCATCTCTGTACTGATACAACC |  |  |
| CAGL0F01419g | KA5 for | GCAGGTGAA*ATG*GTGCTAGTGCTCG | 808bp | F, 145814-146621 |
|  | KA6 rev | CTTCTGAGCATAGAGAAGAAGCAATGCCC |  |  |
| CAGL0F02717g | KA7 for | GGTGTCACCAGAGAGGACTTCGC | 860bp | F, 262174- 263033 |
|  | KA8 rev | GCTGCACCTCTGAAATAGAAAGTGTCAGC |  |  |
| CAGL0J00363g | KA9 for | GTGCATTGTCTGCCAATGAGCAAACAGAG | 811bp | J, 27691- 28501 |
|  | KA10 rev | CCCATCATTGGATCTCTCAACAGCAGAC |  |  |
| CAGL0B02343g | KA11 For | CCGGAAGAGTGGAACTGATAGTGTACAG | 855 | B, 222878-223732 |
|  | KA12 Rev | GCAGAGTCCCAGCCATCTATTGGTG |  |  |

**Table S3.** Quantification of the expression potential of putative resistance genes by RT-qPCR in different *C. glabrata* strains carrying small chromosomes. Note that 1.000 is a relative level found in the standard. The relative gene expression was determined twice and

the values shown as 1^st^ and 2^nd^ experiment (details can be found in Materials and Methods)

| **Strain Number** | **Gene ID** | | | | | | | | | | | |
| --- | --- | --- | --- | --- | --- | --- | --- | --- | --- | --- | --- | --- |
|  | **CAGL0D03674g** | | **CAGL0F01419g** | | **CAGL0F02717g** | | **CAGL0G00242g** | | **CAGL0J00363g** | | **CAGL0B02343g** | |
|  | 1^st^ Exp. | 2^nd^ Exp. | 1^st^ Exp. | 2^nd^ Exp. | 1^st^ Exp. | 2^nd^ Exp. | 1^st^ Exp. | 2^nd^ Exp. | 1^st^ Exp. | 2^nd^ Exp. | 1^st^ Exp. | 2^nd^ Exp. |
| **Y1640** | 1.314 | 1.028 | 0.851 | 0.910 | 1.279 | 1.235 | 0.787 | 1.463 | 0.155 | 0.325 | 1.149 | 1.708 |
| **Y1641** | 1.147 | 1.117 | 0.811 | 0.815 | 1.098 | 1.243 | 1.357 | 1.500 | 0.250 | 0.229 | 1.887 | 1.342 |
| **Y1642** | 0.887 | 0.927 | 1.844 | 2.229 | 1.181 | 1.308 | 0.970 | 1.033 | 1.153 | 1.213 | 3.118 | 2.416 |
| **Y1643** | 1.099 | 0.845 | 1.906 | 2.056 | 1.013 | 0.939 | 1.222 | 0.999 | 1.089 | 0.775 | 1.933 | 1.112 |
| **Y1644** | 0.868 | 0.828 | 0.735 | 0.742 | 0.604 | 0.596 | 1.046 | 0.805 | 0.871 | 0.717 | 2.158 | 1.560 |
| **Y1645** | 1.382 | 1.312 | 1.977 | 2.099 | 1.386 | 1.598 | 1.464 | 1.533 | 1.679 | 1.110 | 1.885 | 1.308 |
| **Y1646** | 1.211 | 1.146 | 0.896 | 0.945 | 0.495 | 0.514 | 1.035 | 0.932 | 1.004 | 1.107 | 2.248 | 1.999 |

**Figure S1.** The *C. glabrata* CBS 138 chromosomes and the position of the centromeres (marked in red) and of the hybridization probes (marked in blue) (see also the Table 2) used in the Southern blot analysis. The chromosomes are named from A to M according to the *C. glabrata* nomenclature (<http://cbi.labri.fr/Genolevures/>).


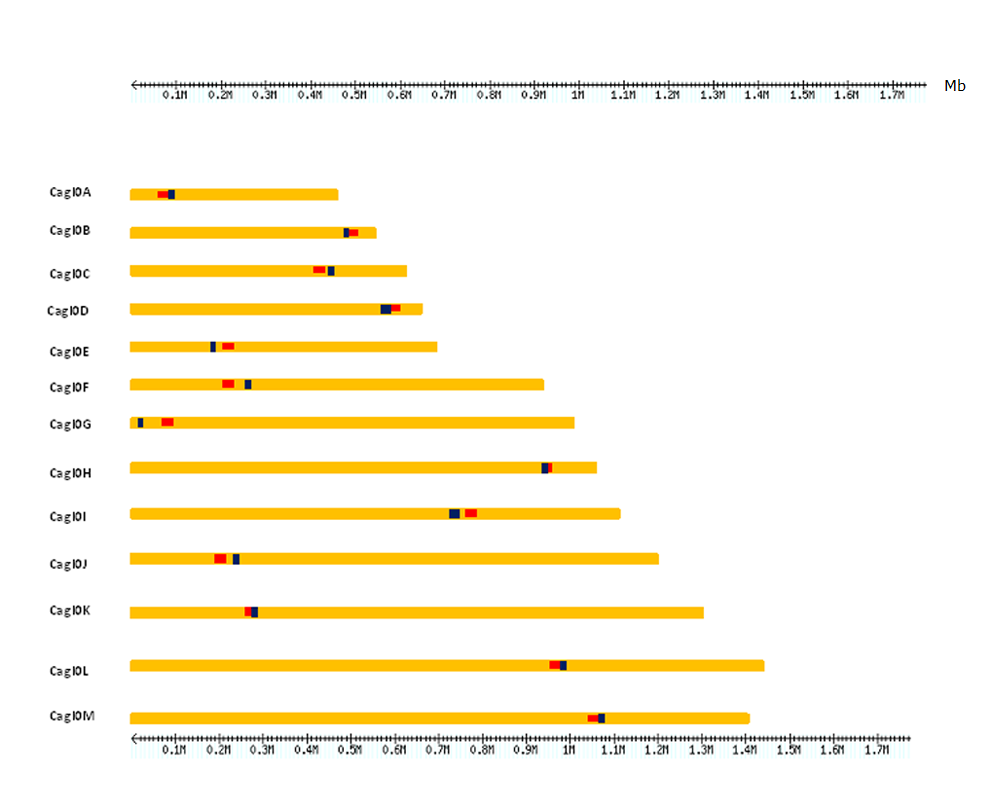


**Figure S2.** Southern blot analysis of the *C. glabrata* clinical isolates with small chromosomes, using chromosome B probe (**Bcl**). **A.** The PFGE gel separation used for hybridization, and CBS 138 is shown as a reference with the chromosome sizes at the left. **B.** Hybridized membrane of the gel shown in A. Note that KA004596 (Y 1646) (arrowed) small chromosome apparently has the same origin as chromosome B. In CBS 138 chromosome B is arrowed as a.





**Figure S3.** Southern blot analysis of *C. glabrata* clinical isolates with small chromosomes using a probe (**Fm2**) originating from chromosome **F**. The Y1643 small chromosome (arrowed) gave a signal with the probe and for this strain only one signal was observed. In Y663 two bands gave a signal, the original chromosome F and the small chromosome. In CBS 138 chromosome F is arrowed as a.





**Figure S4.** Southern blot of *C. glabrata* clinical isolates with small chromosomes using a probe (**Jm2**) from chromosome J. **A.** The PFGE gel (CBS 138 used as a reference and the chromosome sizes shown on the left). **B.** Membrane hybridized with the probe **Jm2** showing a signal with the small chromosome of KA003716 (Y1645) (arrowed). In CBS 138 chromosome J is arrowed as a.


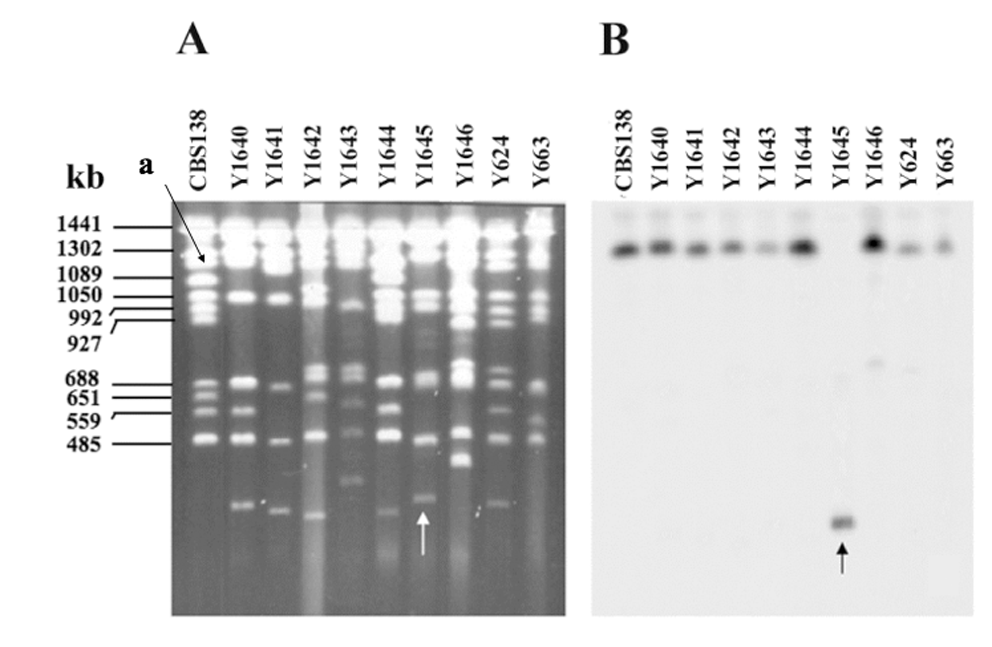


**A**

**Figure S5.** Chromosome separations and Southern blots of *C. glabrata* clinical isolates to detect the presence of the ABC genes (see Table 2) on the small chromosomes, and CBS 138 used as a reference (the chromosome sizes are on the left). **A**. Hybridization performed with the D, F and G probes. **B.** Hybridization performed with the F, J and B probes (and the gene names are shown on the hybridization membranes). The CAGL0D03674g gene was detected on chromosome D, the original chromosome of both Y1642 and Y1644, but not on the small chromosomes. CAGL0F01419g and CAGL0F02717g are present in one copy on the small chromosome of Y1643 (arrowed) but not on the original chromosome F. Both Y1640 and Y1641 have one copy of CAGL0G00242g on their small chromosomes. Y1645 has CAGL0J00363g on its small chromosome, and Y1646 has CAGL0B02343g on its small chromosome.


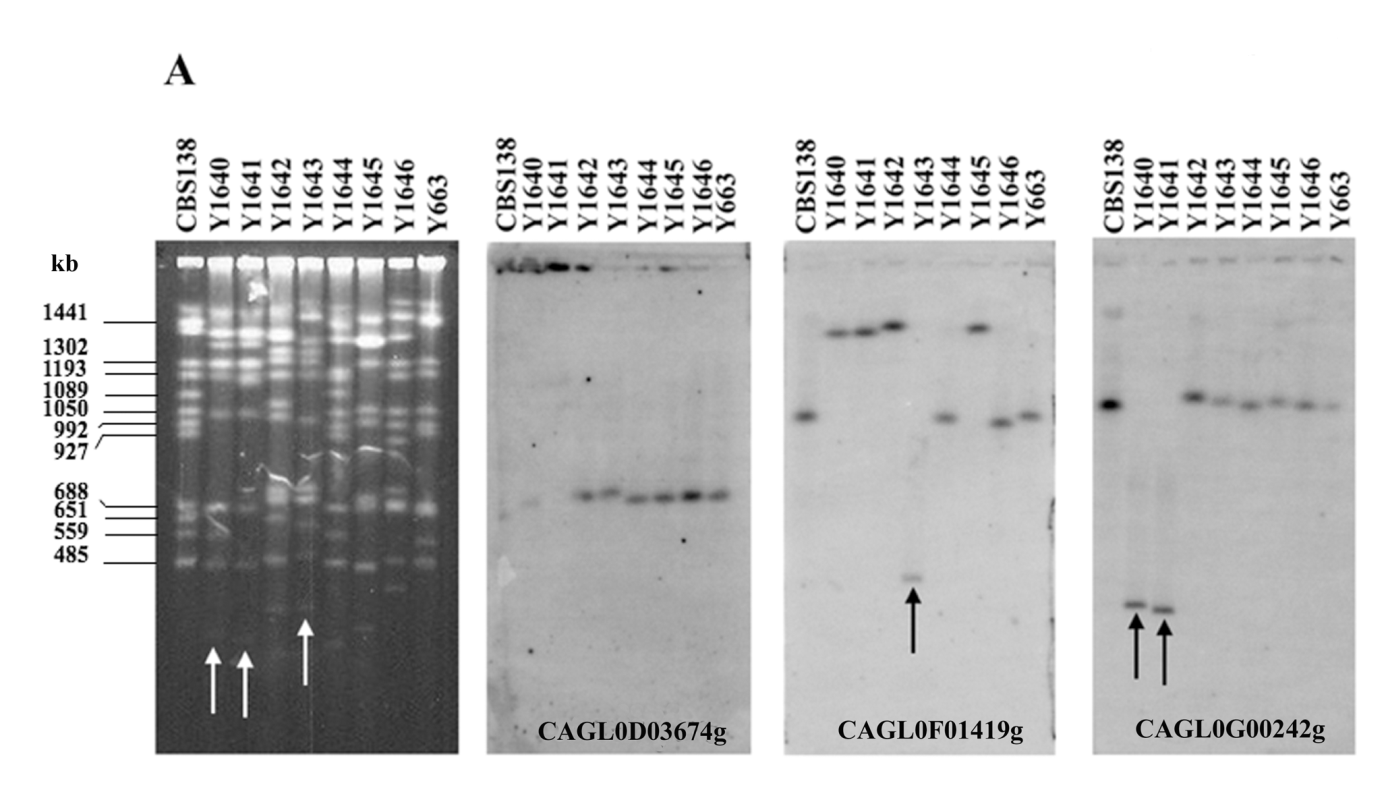


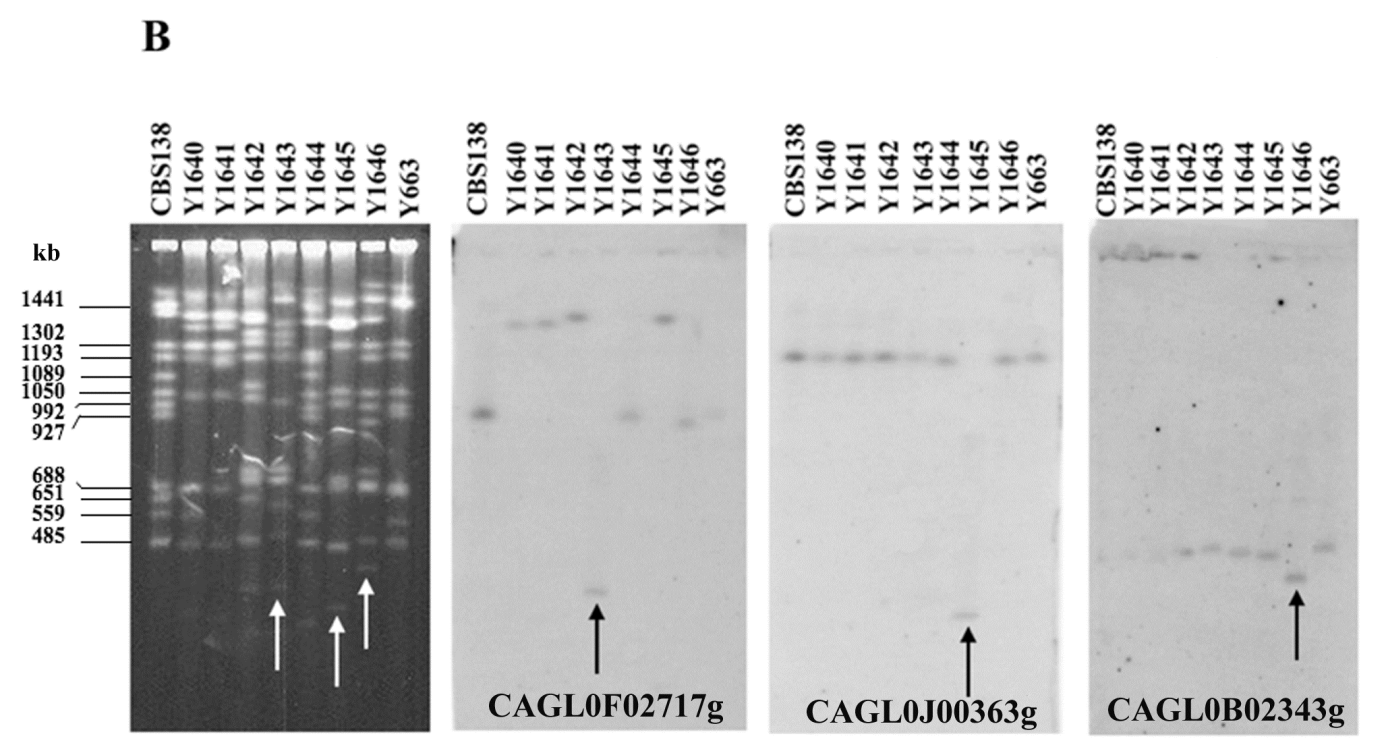


**Figure S6.** A selection of *C. glabrata* clinical isolates with well separated chromosomes is presented in four gels: A, B, C and D. CBS 138 was used as a reference and chromosome sizes are shown on the side of the figures.

**
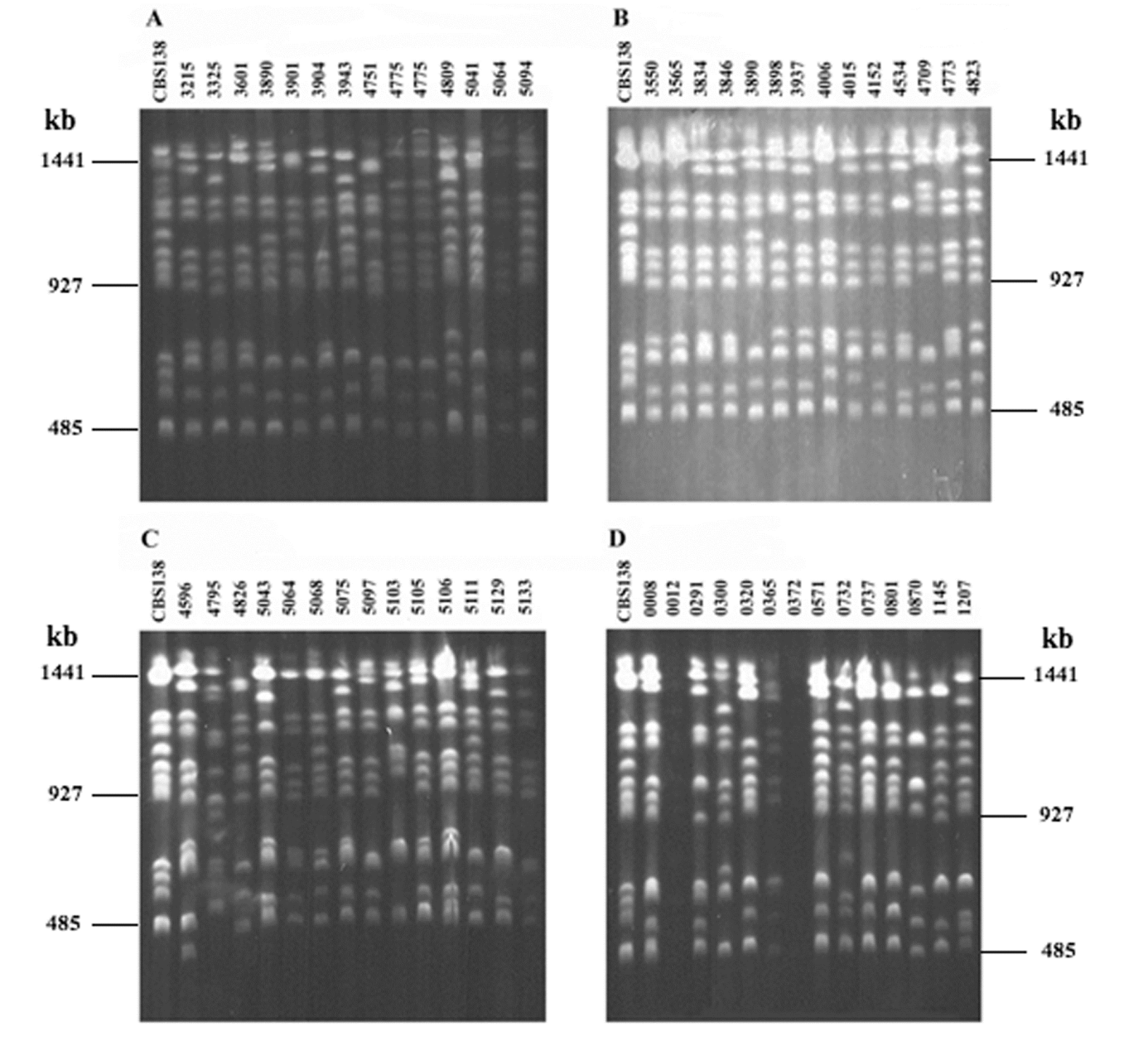
**
